# A Rare Complication of Thymoma: Pure White Cell Aplasia in Good's Syndrome

**DOI:** 10.1155/2019/1024670

**Published:** 2019-10-13

**Authors:** Kim Uy, Elizabeth Levin, Pawel Mroz, Faqian Li, Surbhi Shah

**Affiliations:** ^1^University of Minnesota, Department of Medicine, Division of Hematology, Oncology and Transplantation, Minneapolis, MN, USA; ^2^University of Minnesota, Department of Laboratory Medicine and Pathology, Minneapolis, MN, USA

## Abstract

Pure white cell aplasia (PWCA) is a rare manifestation of thymoma. It is characterized by agranulocytosis with absent myeloid precursors in the bone marrow and normal hematopoiesis for other cell lines. Here we describe a 65-year-old female patient who presented with three days of fever and night sweat. Chest CT revealed an anterior mediastinal mass. A biopsy of the mass confirmed a diagnosis of thymoma mixed type A and B2. The patient developed a severe neutropenia, and her bone marrow revealed significantly decreased neutrophil-lineage cells, rare to absent B cells, and defective T cells, consistent with PWCA. Following thymectomy, a complete resolution of PWCA was achieved via multimodality therapy of intravenous immunoglobulins, granulocyte colony-stimulating factor, and immunosuppressant. This report highlights the care complexity regarding treatment choices and decision to perform thymectomy in patients presenting with PWCA.

## 1. Background

Primary tumors of the thymus gland are rare neoplasm [1]. If occurs, its most common type is benign thymoma. Thymus plays a central role in the development of adaptive immune system, particularly in the maturation process of T lymphocytes. Hence, patients with thymoma often present with a varying type of paraneoplastic syndromes [2, 3]. Good's syndrome or hypogammaglobulinemia is a recognized paraneoplastic syndrome of thymoma, and it is commonly associated with pure red cell aplasia. In contrast, pure white cell aplasia (PWCA) is a rare manifestation, particularly in setting of Good's syndrome. PWCA is characterized by agranulocytosis with absent myeloid precursors in the bone marrow and preserved hematopoiesis for other cell lines [4]. Here we report a thymoma patient presenting with Good's syndrome and PWCA.

## 2. Case Presentation

A previously healthy 65-year-old Caucasian woman presented with three days of fever and night sweats. On exam, she had oral thrush and truncal morbilliform rash. Laboratory workup showed severe neutropenia with WBC 1.5 K/*μ*L, ANC 0 K/*μ*L, and low serum immunoglobulin with IgA 54 mg/dL, IgM 83 mg/dL, and IgG 455 mg/dL. Other standard laboratory workup was unremarkable including detailed infectious and rheumatologic evaluations.

**Figure 1:**
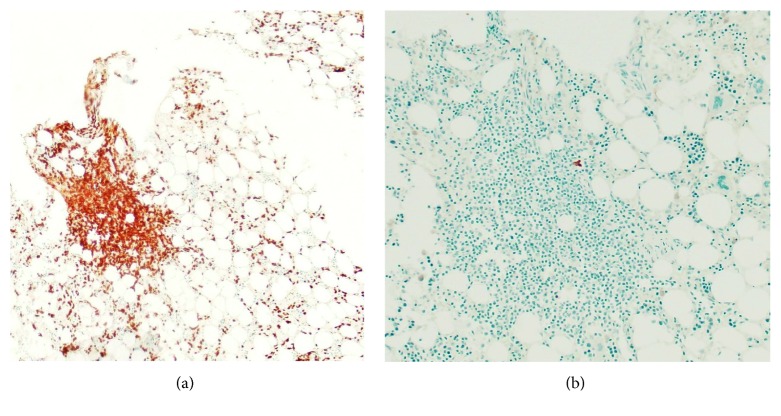
Bone marrow biopsy: bone marrow aspiration of the posterior iliac crest revealed normocellular bone marrow 30–40% with granulocytopenia and increased CD3+ T lymphocytes some in aggregates (a). Flow cytometry showed rare to absent B cells and no aberrant immunophenotype on T cells (b).

A bone marrow biopsy revealed decreased mature granulocytes and granulocytic precursors and increased CD3-positive T lymphocytes in small lymphoid aggregates (Figure 1). Flow cytometry showed rare to absent B cells and no aberrant immunophenotype on T cells. The patient was started on intravenous immunoglobulin (IVIg) and filgrastim without improvement in WBC. A skin biopsy of the rash revealed a dermatitis, suggestive of drug eruption but could not rule out viral exanthem.

**Figure 2:**
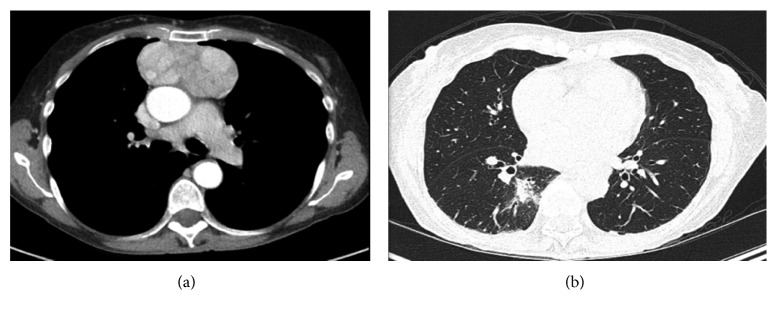
Chest CT: a 4.1 × 8.3 × 7.9 cm mass in the anterior mediastinum with lobulations and dense enhancement (a). Multiple indeterminate nodules throughout the bilateral lungs (b).

**Figure 3:**
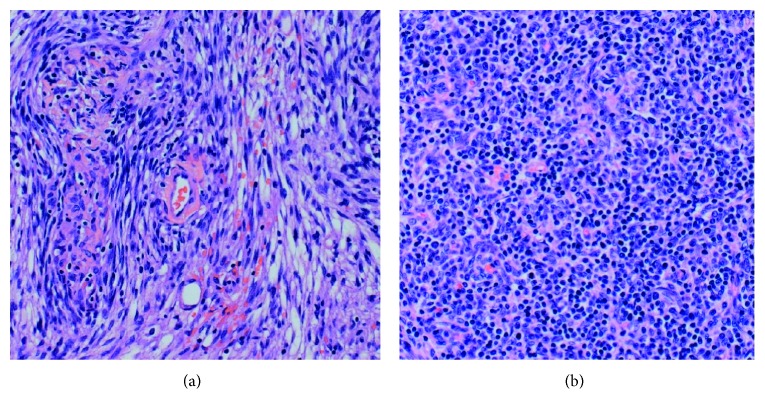
Thymoma mixed type A and B2: grossly and microscopically encapsulated thymoma type A (a) showing spindle cells and type B2 (b) showing mixed epithelial cells and lymphocytes. The lymphocytes are positive for CD3 with rare CD20-positive B cells. These lymphocytes are also positive for TdT and CD1a.

Chest CT scan revealed an 8 cm circumscribed heterogeneously enhancing solid mass in the anterior mediastinum, suspicious for neoplastic process (Figure 2). It also showed multiple indeterminate nodules throughout the bilateral lungs. Given the severe neutropenia of unknown duration, these nodules were concerning for invasive fungal infection. Bronchoalveolar lavage was performed. Fungal cultures were negative after four weeks. Serum beta-D-glucan, galactomannan antigen, Histoplasma antigen, and Cryptococcus antigens were also negative. The patient was empirically started on levofloxacin and voriconazole. A CT-guided mediastinal mass biopsy was pursued, revealing a network of cytokeratin AE1/AE3-positive epithelial cells mixed with CD3+, TdT+, and CD1a+ lymphocytes and rare CD20+ B cells. These were consistent with thymoma type B2. The biopsy also showed spindle cells consistent with thymoma type A (Figure 3). A diagnosis of thymoma mixed type A and B2 was given.

After multidisciplinary discussions, thymectomy was pursued despite concerns of poor wound healing in the setting of severe neutropenia. Pathology on the surgical specimen revealed encapsulated tumor consistent with thymoma, modified Masaoka stage I. Specimen had free tumor margins, and associated lymph nodes were benign. The patient recovered well following thymectomy, and filgrastim was discontinued due to its limited effect during the preoperative period. The patient was continued on prophylactic antibiotics as she remained neutropenic.

On postop follow-up, the patient had persistent neutropenia. Therefore, cyclosporine was initiated for immunomodulation, and filgrastim was reintroduced to boost myeloid stem cells. In one week, the WBC increased to 6.7 from 1.0 K/*μ*L and ANC increased to 2.8 from 0.0 K/*μ*L. However, when granulocyte colony-stimulating factor (G-CSF) was discontinued, WBC decreased to 2.2 K/*μ*L and ANC decreased to 0.3 K/*μ*L over a 3-day period. G-CSF was restarted for an additional one month until cyclosporine reached maintenance levels. In the next 4 months, the patient remained on a maintenance dose of cyclosporine with a target level of 200–400 ng/mL while monitoring toxicity. Subsequently, she was successfully weaned off all immune modulators with self-sustaining WBC after a total of 6-month therapy. Notably, the patient suffered multiple recurrent respiratory infections with CT showing an infiltrate in different lung fields and required short courses of antibiotics throughout her recovery. However, no major infectious complications were observed.

## 3. Discussion

In the United States, the overall incidence of thymoma is 0.13 per 100,000 person-years [1]. Thymoma equally affects males and females with a rising incident in the fourth or fifth decade and peak in the seventh decade of life [2]. Thymomas are neoplasms of thymic epithelial cells typically with mixed cortical and medullary properties. Its classifications are based on the non-neoplastic lymphocyte content and epithelial cell features, which are categorized into type A, AB, B1, B2, and B3 [5]. Surgical removal of thymoma is the mainstay of treatment, particularly for stage I and II thymoma [6].

Approximately, 6–11% of all thymoma cases present with hypogammaglobulinemia or Good's syndrome [2]. Principal findings of Good's syndrome include few or absent B cells, inverted CD4+ : CD8+ T cell ratio, CD4 T-cell lymphopenia, and impaired mitogenic function of T cells. Despite low immunoglobulin synthesis, up to 30% of Good's syndrome presentations are associated with hematologic disorders [2, 3]. The most common associations are pure red cell aplasia and myasthenia gravis [2]. Conversely, pure white cell aplasia (PWCA) in setting of Good's syndrome is a rare occurrence, accounting for 1.1% of patients with thymoma [2]. PWCA is a hematologic disorder characterized by agranulocytosis with absent neutrophil-lineage cells in the bone marrow and preserved erythropoiesis and megakaryocytopoiesis. Most PWCA are associated with type A and mixed type AB thymomas [7]. PWCA has also been found to be associated with drugs, viruses, and other autoimmune disorders [8–11].

Although the etiology of PWCA seen in thymoma remains elusive in the literature, it is consensus that PWCA is of autoimmune origin. Many speculate that presence of thymoma leads to a loss of autoimmune regulator, thus creating a “window of opportunity” for autoimmune disease to develop [3, 12]. Two mechanistic pathways had been proposed to support this theory. The first pathway proposes that dysregulated production of cytokines, possibly mediated by neoplastic thymic stromal cells, influences the growth and differentiation of both the thymus and precursor B cells in the bone marrow [3]. For example, thymic stromal lymphopoietin (TSLP), an IL-7-like cytokine produced mainly by thymic epithelial cells, has wide-ranging impacts on the development of pre-B cells in the bone marrow, as well as regulatory T and Th17 cells in the thymus. Wang et al. found that the levels of TSLP mRNA and protein expression were lower in patients with thymoma-associated myasthenia gravis than in thymoma patients without myasthenia gravis [13]. Other studies of patients with thymic neoplasia also detect autoantibodies against cytokines and a decrease in in vitro cytokine production in response to T-cell simulation [14, 15]. These findings suggested that decrease in the expression of cytokines contribute to T-cell imbalance in the thymus and affect precursor B-cell development in the bone marrow. The second pathway proposes that the abnormal environment of thymoma prevents non-neoplastic T cell to undergo appropriate thymic central tolerance and maturation, thus leading to activation of autoreactive T cells [16]. When these cells escape into the circulation, they could precipitate autoimmune reactions and contribute to abnormal activation of B cells, yielding lymphocytic- and antibody-mediated toxicities to myelomonocytic precursor cells [3]. Interestingly, our patient's bone marrow biopsy showed rare to absent B cells, lack of aberrant immunophenotype on T cells, and agranulocytosis in absence of positive rheumatological tests. These findings suggest that potential inhibitory factors diminish the growth of progenitor cells involving myelocyte and lymphoid lineages.

This case highlights the care complexity regarding treatment choices in patients presenting with PWCA, particularly surgical intervention in the setting of neutropenia and concerns for wound healing. While thymectomy does not necessarily reverse the immunological abnormalities in Good's syndrome, some have observed clinical improvement following resection [17]. Our case demonstrated that while G-CSF and IVIg were completely ineffective prior to thymectomy, the bone marrow was responsive postoperatively. With regards to immunomodulatory agents, the repertoire is broad due to limited literature describing the use of chemotherapeutic agents [18]. In this presented case, cyclosporine treatment at minimal doses was used with complete resolution of PCWA. Patients with PCWA and Good's syndrome are at increased risk of fatal infections due to concurrent leukopenia and immunodeficiency, thus suggesting the importance of supportive care with antibiotics and IVIg. Lastly, similar to other paraneoplastic presentations of thymoma these patients run a risk of recurrence; thereby, they should remain on surveillance.

## References

[1] Engels E. A. (2010). Epidemiology of thymoma and associated malignancies. *Journal of Thoracic Oncology*.

[2] Kelesidis T., Yang O. (2010). Good’s syndrome remains a mystery after 55 years: a systematic review of the scientific evidence. *Clinical Immunology*.

[3] Kelleher P., Misbah S. A. (2003). What is good’s syndrome? Immunological abnormalities in patients with thymoma. *Journal of Clinical Pathology*.

[4] Degos L., Faille A., Housset M., Boumsell L., Rabian C., Parames T. (1982). Syndrome of neutrophil agranulocytosis, hypogammaglobulinemia, and thymoma. *Blood*.

[5] Dzhagalov I., Phee H. (2012). How to find your way through the thymus: a practical guide for aspiring T cells. *Cellular and Molecular Life Sciences*.

[6] Detterbeck F. C. (2010). Evaluation and treatment of stage I and II thymoma. *Journal of Thoracic Oncology*.

[7] Dong J.-P., Gao W., Teng G.-G., Tian Y., Wang H.-H. (2017). Characteristics of goodʼs syndrome in China. *Chinese Medical Journal*.

[8] Akinosoglou K., Melachrinou M., Siagris D. (2014). Good’s syndrome and pure white cell aplasia complicated by cryptococcus infection: a case report and review of the literature. *Journal of Clinical Immunology*.

[9] Tamura H., Okamoto M., Yamashita T. (2007). Pure white cell aplasia: report of the first case associated with primary biliary cirrhosis. *International Journal of Hematology*.

[10] Frattini F., Crestani S., Vescovi P. P., Franchini M. (2013). Pure white cell aplasia induced by mesalazine in a patient with ulcerative colitis. *Hematology*.

[11] Fumeaux Z., Beris P., Borisch B. (2003). Complete remission of pure white cell aplasia associated with thymoma, autoimmune thyroiditis and type 1 diabetes. *European Journal of Haematology*.

[12] Cheng M., Anderson M. S. (2018). Thymic tolerance as a key brake on autoimmunity. *Nature Immunology*.

[13] Wang Z., Chen Y., Xu S. (2015). Aberrant decrease of microRNA19b regulates TSLP expression and contributes to Th17 cells development in myasthenia gravis related thymomas. *Journal of Neuroimmunology*.

[14] Raschal S., Siegel J., Huml J., Richmond G. (1997). Hypogammaglobulinemia and anemia 18 years after thymoma resection. *Journal of Allergy and Clinical Immunology*.

[15] Burbelo P. D., Browne S. K., Sampaio E. P. (2010). Anti-cytokine autoantibodies are associated with opportunistic infection in patients with thymic neoplasia. *Blood*.

[16] Khawaja M. R., Nelson R. P., Miller N. (2012). Immune-mediated diseases and immunodeficiencies associated with thymic epithelial neoplasms. *Journal of Clinical Immunology*.

[17] Jansen A., van Deuren M., Miller J. (2016). Prognosis of good syndrome: mortality and morbidity of thymoma associated immunodeficiency in perspective. *Clinical Immunology*.

[18] Jethava Y., Alamelu J., Rangarajan S., Lang-Lazdunski L., van der Walt J., Fields P. (2011). Acquired agranulocytosis and factor XI deficiency in association with thymoma. *Journal of Clinical Oncology*.

